# Game theory in biology: 50 years and onwards

**DOI:** 10.1098/rstb.2021.0509

**Published:** 2023-05-08

**Authors:** Olof Leimar, John M. McNamara

**Affiliations:** ^1^ Department of Zoology, Stockholm University, Stockholm 106 91, Sweden; ^2^ School of Mathematics, University of Bristol, Bristol BS8 1UG, UK

**Keywords:** cooperation and conflict, animal contests, reciprocity, pseudo-reciprocity, biological markets

## Abstract

Game theory in biology gained prominence 50 years ago, when Maynard Smith & Price formulated the concept of an evolutionarily stable strategy (ESS). Their aim was to explain why conflicts between animals of the same species usually are of a ‘limited war’ type, not causing serious injury. They emphasized that game theory is an alternative to previous ideas about group selection, which were used by ethologists to explain limited aggression. Subsequently, the ESS concept was applied to many phenomena with frequency dependence in the evolutionary success of strategies, including sex allocation, alternative mating types, contest behaviour and signalling, cooperation, and parental care. Both the analyses of signalling and cooperation were inspired by similar problems in economics and attracted much attention in biology. Here we give a perspective on which of the ambitions in the field have been achieved, with a focus on contest behaviour and cooperation. We evaluate whether the game-theoretical study of the evolution of cooperation has measured up to expectations in explaining the behaviour of non-human animals. We also point to potentially fruitful directions for the field, and emphasize the importance of incorporating realistic behavioural mechanisms into models.

This article is part of the theme issue ‘Half a century of evolutionary games: a synthesis of theory, application and future directions’.

## 1. Introduction

Counting from the 1973 seminal article by Maynard Smith & Price ([1], henceforth MSP73), game theory in biology is now 50 years old. The theory was inspired by ideas and analyses in economics and other social sciences, and emerged from the late 1950s onwards. The theory of sex allocation [2], which in principle is a highly successful application of game theory, has a considerably longer history. It originated in works by Darwin and Düsing in the nineteenth century [3–5], independently of game theory. Fisher [6] provided an influential account in 1930, which was further refined by Shaw & Mohler [7] in the early 1950s. Hamilton [8] extended previous analyses in fundamental ways in 1967, and mentioned game theory as a possible framework.

**Figure 1.**
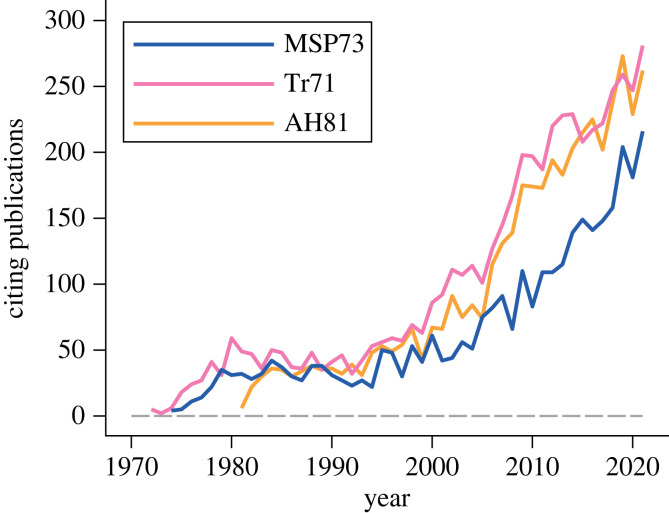
Number of papers per year citing each of three classical papers in game theory in biology: MSP73 [1], Tr71 [11] and AH81 [12]. Citation data derived from Clarivate Web of Science. Copyright © Clarivate 2022.

Our aim here is to provide a perspective on the successes and challenges of game theory in biology. Game theory is important for all areas of biology where there is frequency dependence in the reproductive success of phenotypes, so that the best phenotype of an individual depends on the phenotype of others. In addition to sex allocation, other examples from life-history evolution are dispersal morphs [9] and alternative reproductive phenotypes [10]. We do not cover these in any detail, but instead focus on a smaller set of basic questions about conflict and cooperation in the evolution of behaviour that were introduced in early influential game-theory articles. These are MSP73 [1], which studied the evolution of animal contest behaviour, the 1971 article by Trivers ([11], henceforth Tr71), and the 1981 article by Axelrod & Hamilton ([12], henceforth AH81). The latter two studied the evolution of cooperation. As seen in figure 1, these have had a notable impact on biology.

After briefly summarizing the early history of game theory in biology, we cover both the broader impact of the articles and, in particular, their specific biological questions and predictions. We review how game-theory models and observations have progressed in studying and resolving the questions over a 50-year period. Finally, while the social sciences have historically had a strong influence on game theory in biology, we emphasize the need for biology to develop its own style of explanations and modelling approaches. We argue that, among these, incorporating behavioural mechanisms into game-theory models is a promising future direction. This approach can increase biological realism and allow closer contact between theory and observation.

## 2. Early history of game theory in biology

The first direct appeal to game theory as a way to understand biological phenomena may have been by Fisher [13] in 1958, in a discussion of genetic polymorphism. He argued that certain predator–prey interactions, such as those between mimetic butterflies and bird predators, could be understood using game theory, of a kind he had previously used himself [14] when analysing strategies in a card game called ‘Le Her’. Fisher’s suggestion seems not to have had a major influence on biology, although the importance of frequency dependence for the evolution of polymorphic Batesian mimicry is widely recognized [15,16].

An ambitious attempt to use game theory to describe adaptation to variable environments was developed in 1961 by Lewontin [17], using the idea that populations can be viewed as playing games against ‘nature’ (meaning local environmental conditions). As pointed out by Maynard Smith [18–20], it seems that Lewontin implicitly assumed group or species selection in developing his theory. Partly for this reason, the idea of viewing local adaptation as a game populations play against ‘nature’ is no longer pursued in biology.

As mentioned, Hamilton’s 1967 article [8] made fundamental contributions to the study of sex allocation, also taking into account mating between relatives (local mate competition). It also seems to be the first paper that directly mentioned game theory (and so-called minimax strategies) in connection with sex allocation. Still, Hamilton did not cite any specific papers on game theory.

## 3. Animal contests

### (a) Maynard Smith & Price (1973)

MSP73 [1] has become a focal point for game theory in biology. A major reason for its impact (figure 1) is that it introduced the concept of an evolutionarily stable strategy (ESS). This is an elaboration of the Nash equilibrium (box 1) and is central to the study of interactions with frequency dependence in the success of strategies. MSP73 dealt with strategies consisting of either discrete actions or a probabilistic mixture of different actions. Often in game theory one wants to study strategies that are represented as quantitative traits, and for this one needs a condition for whether gradual evolution converges towards an equilibrium (box 1).

Box 1. **Evolutionary stability.**

How do we expect natural selection to shape behavioural strategies when there is frequency dependence? In evolutionary game theory, the basic approach is to seek stable endpoints of the evolutionary process, rather than following an evolutionary trajectory under detailed assumptions about the underlying genetics.

It is easiest to characterize stable endpoints at which the population is monomorphic. Suppose that almost all population members follow the same strategy *x*. Then we refer to *x* as the resident strategy. Let *x*′ be a rare mutant strategy in this population, and denote the fitness of this mutant strategy by *W*(*x*′, *x*). Evolutionary game theory has introduced two standard criteria that must both be met for a strategy x* to be considered as a stable endpoint.

1. *If the resident population is* x* *then no single mutant strategy can invade under the action of natural selection*. In fitness terms, a necessary condition is that
  3.1 $$W(x^{\prime },x^{*})\leq W(x^{*},x^{*})\text{ for all mutants }x^{\prime }.$$
  This is the familiar Nash equilibrium condition of economic game theory. In order to deal with mutants that are equally fit as residents, MSP73 [1] strengthened this with a second condition demanding that
  3.2 $$\text{if }W(x^{\prime },x^{*})=W(x^{*},x^{*})\text{ for some }x^{\prime }\neq x^{*}\text{ then }W(x^{\prime },x^{\prime })<W(x^{*},x^{\prime }).$$
  A Nash equilibrium strategy x* that satisfies this second condition is referred to as an ESS.
2. *If the resident population is perturbed away from* x* then natural selection will lead back to x*. When the trait *x* is one-dimensional, this will happen if the selection gradient *F*(*x*) near *x* = x* satisfies
  3.3 $$F(x)>0\text{ for }x<x^{*}\text{ and }F(x)<0\text{ for }x>x^{*},\text{where }F(x)=\frac{\partial W}{\partial x^{\prime }}(x,x).$$
  The strategy x* is then said to be convergence stable [21,22].

If x* satisfies both criteria above we can regard x* as a realistic endpoint of the evolutionary process. Note that the first criterion (ESS) is a global condition, whereas the second (convergence stability) is a local condition.

Some of the impact of MSP73 also came from its emphasis on individual selection in game theory, thus presenting an alternative to ideas about group and species selection that were widespread at the time [23,24]. Lorenz [24] argued that animals limit their aggression towards conspecifics in order not to endanger the survival of the species, while MSP73 put forward game theory as an alternative.

The main ingredient in MSP73 was a model of the evolution of non-dangerous contest behaviours, such as threat displays, ‘ritualized’ aggression, and pushing contests. The Hawk–Dove game [20,25] is often mentioned as the model studied in MSP73, but this is strictly speaking not correct. Instead, the main biological argument was that a strategy referred to as ‘retaliator’ (starting with non-dangerous display behaviour and escalating to dangerous fighting if the opponent uses dangerous behaviour) is an ESS, and that this strategy explains limited aggression in animal contests.

The ‘retaliator’ strategy in MSP73 has an interesting history. It seems that it was inspired by game-theory analyses by social scientists studying conflicts between nations during the Cold War, coming up with the idea that mutual deterrence through threats of retaliation could prevent escalation to a dangerous and costly war. An example would be work by Schelling [26], who was awarded the 2005 Nobel Memorial Prize in Economic Sciences for such analyses. Price was well informed about Cold-War theorizing, and even had a contract on a never-finished book about the Cold War, as is described in a biography [27]. Price had broad interests and, although not primarily a biologist, in 1968 he submitted a manuscript on game theory of animal contests to the journal *Nature* (these events were researched by Oren Harman [27,28]). The title of the manuscript was ‘Antlers, intraspecific combat, and altruism’. The text is not readily available, but it seems [28] to contain the essential reasoning in MSP73. The manuscript was reviewed by Maynard Smith and, although it was rejected for being too long for the journal (as described by Maynard Smith [20]), he was impressed by it. In fact, Maynard Smith’s first work on game theory of animal contests [18] started out describing antler fights in deer, in a manner that appears to be inspired by Price’s manuscript (see also the description in [20]).

Surprisingly, there is no citation of works on game theory in MSP73, nor is there any mention of Cold-War theories of conflict. The main analysis is instead a computer simulation, where a few specific strategies (referred to as ‘mouse’, ‘hawk’, ‘bully’, ‘retaliator’ and ‘prober–retaliator’) were compared, with the conclusion that ‘retaliator’ is an ESS (the numerical correctness of this conclusion was quickly questioned [29]). Each strategy followed a simple rule about when to use ‘conventional’ versus ‘dangerous’ behaviour. Apart from the possible value of this game-theory analysis, there was a general discussion about animal contests in MSP73 and in the preceding work mentioned above. Maynard Smith & Price discussed various observed aggressive behaviours, such as pushing contests in the antler fights of red deer males, and signalling through threat displays, such as 'stomping' and bellowing, and they noted that there is likely to be a high positive correlation between the prowess in such activities and the fighting ability in an escalated contest. This was not part of their game-theory analysis but fits well with earlier ideas put forward by ethologists (e.g.[30]) and points to questions that subsequently became central to game theory of animal contests.

### (b) Successes and challenges of game theory of animal contests

The general ideas discussed in MSP73 [1] gave inspiration to much game-theory modelling and experiments. New topics were also subsequently introduced, for instance the life-history context of conflicts, owner–intruder contests over territories, and the formation of dominance hierarchies in social groups.

#### (i) Assessment

A major step towards a closer link between observations of animal contests and game-theory models was taken by Parker [31]. He accepted the idea of a retaliator strategy from MSP73, but argued that behaviours shown during a ‘conventional’ phase of a contest (e.g. pushing in antler fighting) will evolve to assess relative fighting ability, and thus indicate which contestant would prevail in an escalated contest. He noted that a proportion of contests might escalate to dangerous fighting; this might happen if contestants are similar enough that imperfect assessment through non-dangerous behaviour fails to reveal a decisive advantage for either of them. His conclusions were based on game-theory reasoning and discussion of observations of animal contest, although Parker [31] did not introduce any specific mechanism of assessment. Subsequent fieldwork and experiments further strengthened Parker’s views on assessment through non-dangerous aggressive behaviour [32–34].

The evolution of weapons, such as horns in ungulates, throws light on the relative importance of assessment versus dangerous retaliation and escalation in many groups of animals [35,36]. As summarized by Emlen [36, p. 405]: ‘Weapons begin as relatively small and very dangerous traits (e.g., short, sharp horns, fangs, tusks). Later versions tend to be much larger, more complex, and in particular, more likely to serve as indicators of status assessed by rival males’. Thus, it seems that evolutionary change of weapons in many species has progressed from causing damage towards being used for display and controlled aggressive interactions, such as head butting and pushing contests.

The sequential assessment game [37] implemented a mechanism of assessment of relative fighting ability, consisting of repeated sampling with errors of observation, similar to how one estimates a mean in statistical sampling. The model was adapted to a number of different situations, such as owner–intruder interactions [38], differences between contestants in the value of winning [39] and dangerous fighting [40,41]. There was also a tentative application of the model to situations with several non-dangerous behaviours [42], including a comparison to experimental data from contests between males of the cichlid fish *Nannacara anomala*. The analysis showed qualitative agreement between model predictions and observations, in particular concerning a division of contests into discrete phases, with less costly but less informative display behaviours appearing early in a fight, and more costly and informative behaviours appearing later, in longer contests between opponents with similar fighting abilities [42]. This is broadly in agreement with much observation of animal contests, for instance the ‘roaring’, ‘parallel walking’ and ‘antler fights’ of red deer males [33], but there are also species where there is no clear division of contests into phases [43,44].

The sequential assessment model assumes mutual assessment, in which individuals gain information of their own fighting ability in relation to an opponent. The assumption has been criticized as being unrealistic for many contests [45,46], where individuals instead might gain more information specifically about their own, or about their opponent’s, strength and condition during interactions. Game-theory models have not explored this issue, nor have they conclusively resolved the question of when and why contests are divided into clear phases. Thus, in spite of much success for the idea of assessment in contests, the field is still open for further work.

#### (ii) Signalling in contests

Some aggressive display behaviours, like the roaring of red deer males, give reliable information about an individual’s size or strength, which is a well established and supported idea [25,32,33,47–49]. The basic reasoning is that it would be too costly, or even impossible, for weak individuals to signal that they are strong, which is related to ideas about signalling in the social sciences [50] and to handicap signalling in biology [51–53].

There is less agreement on whether signals of intent or motivation (e.g. an individual’s value of winning) can be evolutionarily stable. The original game-theory-inspired reasoning was that communication of intent or motivation should not be part of an ESS [19,25,47–49], because a less motivated individual could ‘cheat’ by signalling that it is highly motivated, in order to discourage opponents, thereby undermining the reliability of the signal. A contrasting analysis was developed by Enquist [54], concluding that a less motivated individual might lose from signalling high motivation, because the individual might face a risk of being attacked by a highly motivated opponent.

Many animals have a repertoire of threat displays, and birds are among the most studied groups. Observations support the idea that more motivated individuals tend to use more high-level threats [55], thus in principle providing information about their motivation or possibly their intent. Still, there is no detailed correspondence between game-theory models and much observed behaviour, which can contain long sequences of different threat displays [55]. Also, the temporal relation of threats and attacks during interactions need not show a clear pattern of early threats followed by later attacks [56]. This, together with the question of why there are several different threat displays [57], means that the nature of threat signalling remains a challenge for game theory in biology.

#### (iii) Dangerous contests and life-history context

The fitness value of winning a contest can influence the ESS of a game. For instance, for a symmetric Hawk–Dove game [20,49] the ESS is pure Hawk if the value of winning is greater than the cost of injury in a Hawk–Hawk fight, but is a mixed (randomized) strategy for lower values of winning. Estimates of fitness payoffs from experiments and field data have also been used to explain observed behaviour [58].

A major game-theory insight is that dangerous contests, including those with fatal fighting, should evolve when the value of a current win is substantial in comparison with any additional future reproductive success [40,59]. In an extreme case where there is no future reproduction for the loser of a contest, there is no incentive for a weaker individual to withdraw, and fatal fighting is the predicted outcome. An example is fighting between newly emerged honeybee queens [60]. At most one of them can inherit the colony, and observations show that they fight to the death [60], even though they are likely to be relatives. Male fig wasps emerging in the same fig engage in dangerous fights [61] (see also [62, ch.13]). A comparison across species shows that there are more severe injuries from male–male fighting when fewer females emerge in a fig [63], i.e. when competition for mating is especially severe. For contests between males of the bowl and doily spider, the parameters of a version of the sequential assessment game with dangerous fighting were successfully fitted to data, including predictions on whether a contest ends with lethal injury or with the giving up of a contestant [41]. Bowl and doily spider males fight over difficult-to-find and therefore high-value female webs.

Overall, limited reproductive prospects beyond the current interaction are characteristic of dangerous contests in nature. This conclusion shows how game theory subsequently modified and clarified the seminal work by MSP73 [1], by essentially resolving the question of when dangerous fighting is expected. It is not threats of retaliation and escalation that are crucial but, rather, the life-history context determines the cost of fighting.

The evolution of animal weapons further underscores the point. Thus, an evolution of weapons towards a function in display and assessment [36], as mentioned above, is not found in species with truly dangerous contests. Instead, the stings of honeybee queens and the mandibles of male fig wasps have evolved to be efficient at damaging or even killing opponents.

#### (iv) Owner–intruder contests

There is sometimes a clear-cut asymmetry between contestants. The distinction between the ‘owner’ of a territory or other resource and an ‘intruder’ is the most studied case. There are several game-theory analyses finding ESSs for games with such role asymmetries [25,38,64,65]. An early striking result was that contests might be settled without fighting, with one role conventionally winning [25]. So, an ‘intruder’ discovering that a resource already has an ‘owner’ might simply withdraw.

This possibility was tested in a field experiment with males of the speckled wood butterfly (*Pararge aegeria*) [66]. These males sometimes perch on vegetation in pools of sunlight on the forest floor (sunspot territories) and look for and pursue flying females. If another male appears, an aerial contest can occur. In the experiment, an owner was captured in a butterfly net and then released when another male had arrived [66]. The result was that the new owner invariably won the resulting brief interaction. This seeming success for a game-theory prediction was, however, not confirmed in subsequent work. Instead, field observations on this species showed that when an owner temporarily left the territory and another male moved into the sunspot, the previous owner typically recaptured the territory after an aerial contest [67]. Also, an experiment in a large outdoor cage showed that a speckled wood male’s recent experience of encountering females near a territory strongly influences his chances of winning a contest, by making him persist for longer [68]. This is qualitatively in accordance with an ESS of the so-called war-of-attrition with random rewards [69].

Overall, owners tend to win contests against intruders, and the main explanation is likely to be that owners either have higher fighting ability or are more motivated, in the sense of estimating a higher value of winning [38].

#### (v) Social dominance and winner–loser effects

In social groups, conflicts over resources like mating or foraging opportunities are often resolved through social dominance. There can be fighting over dominance positions during hierarchy formation, but once dominance relations are established, conflicts can be settled without additional fighting. Social dominance throws light on animal cognition, as hierarchy formation is potentially more complex than deciding the outcome of a single contest. It can, for instance involve individual recognition, winner–loser effects, and bystander effects. These possibilities make game-theory modelling of social dominance challenging, which may explain why the field has taken long to develop [70].

One modelling possibility is to assume that there is independent assessment of relative fighting ability in each contest, for instance in some version of the Hawk–Dove game [64,71], and to investigate if pairwise wins and losses result in dominance positions that increase with fighting ability, forming a linear hierarchy [72]. Another approach is to assume that winner–loser effects are important for hierarchy formation [73–75]. Winner (loser) effects occur when an individual’s win (loss) in a contest increases (decreases) its tendency to win (lose) additional contests against different, not previously encountered individuals [76]. Winner–loser effects are commonly observed [77] but are not universal. There can also be bystander effects, which presuppose individual recognition and occur when individuals observe and are influenced by the outcome of contests between other individuals [78]. A recent approach that incorporates these various effects into game-theory models is to assume that individuals use behavioural mechanisms similar to reinforcement learning when forming hierarchies [79,80]. This has, for instance, the potential to explain when winner–loser effects are expected [81].

## 4. The evolution of cooperation

Game-theoretical work on the evolution of cooperation started some 50 years ago, around the same time as the application of game theory to animal contests. Many of the ideas were taken over from analyses of human cooperation in the social sciences. The 1965 book by Rapoport & Chammah [82] is an example of a major influence. It contains analyses of experiments with pairs of university students playing many rounds of the Prisoner’s Dilemma game (PDG). The book briefly mentions the tit-for-tat strategy (i.e. in a game with options C and D, start with C and then use the option the partner used in the previous round), although without discussing whether the experimental data corresponded to tit-for-tat, or to gradual learning of this strategy.

### (a) The articles by Trivers (1971) and Axelrod & Hamilton (1981)

Soon after publication, both Tr71 [11] and AH81 [12] attracted attention, and the impact has continued and even accelerated in recent years (figure 1). A likely reason is an intense general interest in the phenomenon of cooperation, not least in the study of human behaviour, combined with many possibilities for theoretical and experimental elaboration of ideas about cooperative interactions. Our focus here is to summarize the main biological issues raised in Tr71 and AH81, and to comment on the support they provided for their conclusions.

The terms reciprocal altruism and reciprocity were used in Tr71 to mean reciprocal exchanges in which individuals respond to a partner’s ‘cheating’ (the partner not giving help) by withholding help, but Tr71 also included other forms of helping under these headings. For instance, there is the statement that ‘Reciprocal altruism can also be viewed as a symbiosis, each partner helping the other while he helps himself’, with the comment that an important characteristic of such exchanges is that there is a time lag between an individual’s investment and the return benefit [11, p. 39]. The non-human examples in Tr71 (cleaning mutualism and warning calls) were in fact of this kind and could, using a common terminology, be referred to as instances of pseudo-reciprocity (investment in by-product benefits; see below). The terminology in Tr71 might be reasonable, but it has not prevailed. Here we use reciprocity in a strict sense, signifying an ongoing reciprocal exchange between two individuals in which there is a potential immediate benefit of cheating and the exchange is regulated by the withholding of help to cheaters, in a qualitatively similar way as for the tit-for-tat strategy. Pseudo-reciprocity is then an alternative to reciprocity in explaining cooperation.

There was no detailed game-theory analysis in Tr71, but there was a comparison of the payoff of reciprocal altruism with that of defection (not giving help) when a number of rounds of the PDG is played. Much of the presentation consisted of detailed discussions of three potential examples, one being cleaning mutualism in fish, another warning calls in birds, and the third human reciprocal altruism, with an emphasis on the emotional states that might underpin this behaviour.

Concerning cleaning mutualism, for instance between a cleaner wrasse and a client grouper, Tr71 stated that the cleaning interaction itself is mutually beneficial and needs no further explanation. Instead the point was made that predatory client fish, like the grouper, refrain from trying to consume the cleaner once the cleaning is over, and that the evolutionary explanation for this is that the client might need cleaning services in the future. From what is known about cleaning mutualism [83], this interpretation is reasonable. Still, refraining from trying to eat the cleaner is not a case of reciprocity in the strict sense.

Concerning warning calls (alarm calls when a predator is detected), the point was made that giving the call might involve some risk of being attacked by the predator, but the call can also have the effect of making the predator fail to catch prey, therefore perhaps learning not to return to the local area, which is beneficial for the individual giving the call. This is not the only possible explanation of warning calls (in those species that have specialized calls of this kind), but the idea has gained at least some support in subsequent observation and modelling [84,85]. Nevertheless, the explanation is not a case of reciprocity in the strict sense. The somewhat surprising conclusion, also noted previously [86], is that Tr71 did not claim that reciprocity in the strict sense is important in non-human animals, but rather that humans often show this form of cooperative behaviour.

The game-theoretical reasoning in AH81 [12] consisted of discussion of the results of computer tournaments, in which many rounds of the PDG were played between various strategies. The tournaments were conducted by Axelrod, who invited a number of scientists to submit entries (strategies; see [87] for details). There were two tournaments, and in both of them the strategy tit-for-tat, submitted by Rapoport, was considered the winner.

Concerning possible biological applications, AH81 focused on the requirement that, for tit-for-tat to work, interacting organisms must meet repeatedly. A strikingly wide range of species and interactions was mentioned, from cleaning and many other mutualistic interactions to birds in neighbouring territories, but no specific empirical evidence or suggestions of decisive observations or experiments were put forward. An interpretation is that the impact of AH81 owes more to what it implicitly suggested and alluded to than to what it actually demonstrated.

### (b) Successes and challenges of game theory of cooperative behaviour

Given its origin in the social sciences, it is only natural that much debate on the repeated PDG is found in fields like economics, social psychology, and philosophy. Opinions vary, but there is a tendency towards criticism of Axelrod’s work (see, for example, the 2015 edited volume by Peterson [88]). In biology, there is a similar diversity of views on the importance of reciprocity for cooperation in non-human animals and other organisms.

Here we discuss the successes and challenges of game theory of cooperation, with some focus on interactions that could be widespread and significant in the daily lives of organisms. Over the years, a number of valuable ideas about cooperation have been developed and investigated, perhaps the most important being pseudo-reciprocity and market effects from partner switching.

#### (i) Repeated Prisoner’s Dilemma

Either the repeated PDG between two players consists of a finite number of rounds, or there is a given probability to continue to one more round. In the latter case, the number of rounds follows a geometric distribution. It is not obvious that such games correspond to interactions typically occurring in the lives of real organisms [89,90], but they were the prime object of study in AH81 [12] and in much subsequent work, so it is reasonable to summarize where things stand.

Rather soon after AH81, it was noted that tit-for-tat is not an ESS [91], because the strategy fails to satisfy the second condition of the definition (see box 1). As a simple illustration, to always cooperate (always use C) does equally well as tit-for-tat against a tit-for-tat partner, and similarly against an always C partner. One might wonder why, in spite of this, tit-for-tat was successful in the computer tournaments. Computer tournaments need of course not correspond to evolution. There are many additional complications of this approach (see [92] for a discussion), and there is little reason to think that it is of particular relevance to biology.

Using the idea that, realistically, players sometimes make mistakes when executing a strategy, one can find ESSs for the repeated PDG. ‘Contrite tit-for-tat’ [93] and ‘win–stay, lose–shift’ [94] are two much discussed examples. One can, using a mathematically precise concept of evolutionary stability [95], show that there are in fact many relatively simple ESSs [96]. Thus, there are certainly many ESSs for the repeated PDG. Still, while the game-theoretical analysis of these games has been successful, for biology the issue of whether the games correspond to typical interactions in nature remains.

An additional consideration is that, in practice, there is often quite a lot of variation in the traits and strategies of organisms. This can have a strong, qualitative influence on the evolution of cooperation [97]. For instance, if players show notable variation in their strategies, cooperation can evolve for finitely many rounds of the PDG [98], which otherwise so-called backward induction would exclude.

#### (ii) Experimental games with non-human subjects

Empirical studies of human cooperation typically take the form of experimental games. Such studies are likely the inspiration for experimental games with non-human subjects, for instance, by arranging artificial operant environments, either with reward schedules corresponding to a PDG, or where individuals need to coordinate their behaviours to obtain reward. An important proviso for the method is the assumption that learning could be an explanation for cooperative behaviour and that this applies to artificial situations not encountered in the wild.

Quite some time ago, experimental psychologists found that pairs of rats can learn to coordinate their behaviour, for instance taking turns in which one rat sits on a platform to allow the other to feed without getting shocked [99]. In experiments designed to correspond to a repeated PDG, a common finding is that rats to a large extent end up not cooperating [100,101]. There are studies using rats that find more cooperation, with CC responses in almost half the trials (rounds) [102], but the temporal patterning of these responses did not agree with tit-for-tat or with suggested ESSs for the repeated PDG, like the ‘win–stay, lose–shift’ strategy. Other studies also show that rats help and increase their helpfulness somewhat after receiving help [103–105]. These are interesting observations, but their importance for rat behaviour in nature is not known.

An experiment with pairs of blue jays found that the birds do not learn to cooperate when rewards correspond to a PDG, but they learn to cooperate if C gives a higher reward than D irrespective of the partner’s action [106]. Overall, experimental games have delivered interesting results, but their relation to ESSs for the repeated PDG is uncertain.

#### (iii) By-product mutualism and pseudo-reciprocity

Mutualism can be maintained by ‘ordinary selfish behaviour incidentally benefiting neighbours’ [107, p. 19], which can be as simple as individuals, sometimes of different species, joining a flock to reduce their predation risk, at the same time decreasing the predation risk of their neighbours. The term by-product mutualism [108] is used to describe such interactions.

A further possibility is that an individual can increase the by-product benefits it obtains from another by investing in or providing a service for the other. This has been suggested as an explanation for particular interactions, with the discussion of cleaning mutualism in Tr71 perhaps as one example, but it was Connor [109] who emphasized the likely broad importance of the phenomenon and who coined the term pseudo-reciprocity for such interactions. There are many ways in which investments can enhance by-product benefits, with a corresponding rich set of interactions in nature that might be explained in this way [86,109–113]. An important distinction between pseudo-reciprocity and reciprocity is that, for the former, responding to or controlling cheating by a partner is not a primary consideration. The reason is that it is in the immediate interest of a partner to provide by-product benefits.

Most of the conceptual development of ideas about by-product mutualism and pseudo-reciprocity have been in the form of general reasoning about adaptation, rather than explicit game-theory models. There are notable exceptions, for instance a game-theory model about negotiated feeding efforts in bi-parental care [114]. Bi-parental care can be seen as a case of coordinated by-product mutualism, where individuals jointly invest in a ‘project’ that each of them can benefit from. Another example is a model about group augmentation in cooperative breeding [115], showing that helping to raise the offspring of others can be favoured if there are return benefits, like future territorial defence, from the offspring.

#### (iv) Partner switching and biological markets

One type of deviation from the assumptions of a repeated PDG is that individuals can terminate unprofitable interactions and potentially find different partners. Game theory models of such interactions have been analysed [116,117], making the point that partner switching can make cheating more effective, for instance through rapid exploitation of a sequence of partners. This can be counteracted through more effective detection of cheating partners [116] or by imposing costs of switching [117].

The concept of a biological market, developed by Noë & Hammerstein [118,119], further extends these ideas. In a market, there can be interactions between two classes of ‘traders’ (partners), one with offers (investments) and the other responding through by-product benefits. The exchange between partners is similar to pseudo-reciprocity, but with the crucial, market-like element of several offers by partners of one kind and choices between offers by the other, responding partners. Among the examples are mutualisms where ants respond to food rewards offered by a partner organism, as for instance so-called ant plants or various insects offering nutritious secretions. The return benefit from the ants can be protection from enemies, occurring as a by-product of the regular foraging activities of ants. Many types of mutualistic exchanges can be seen as a form of trade. Mycorrhizal symbiosis, in which carbohydrates and mineral nutrients are exchanged between plant roots and fungal hyphae [120,121], is a mutualism of particular importance in terrestrial ecosystems.

Although responding to and punishing cheating are not necessary for a biological market to operate, in practice there are many ways that individuals can extract benefits against their partner’s interest. Cheating in fact occurs regularly in many mutualisms [122], and partners employ various means to counteract and punish cheating. Marine cleaning mutualism is a well-studied example, with both field and experimental work [123,124] and game-theory modelling [125]. Cleaners remove ectoparasites from clients, but prefer to take bites of client skin mucus, to which clients can respond by leaving or chasing a biting cleaner.

Biological markets often show similarities to the markets studied in economics, for instance in the consequences of changes in supply and demand, but the mechanisms regulating exchanges can differ [126], potentially influencing the trade. For instance, game-theoretical modelling of the effect of changing the supply-to-demand ratio for a cleaning mutualism, including specific assumptions about cleaner optimal foraging for ectoparasites on clients, showed that cleaning duration and service quality could either decrease or increase when demand for cleaning service increased [127]. From ideas about supply and demand in economics, one would expect that increasing demand should lower service quality. Data from a field experiment failed to confirm this [127], which might be explained by the specific mechanisms regulating the exchange. Overall, the combination of game-theory modelling and empirical observation has contributed significantly to the current understanding of mutualistic interactions.

#### (v) Reciprocity versus alternatives

Over the years, there has been much debate about whether there is reciprocity (in the strict sense) in non-human animals, or if observed cooperation instead has different explanations, and we give a few examples. The issue is not settled, but there is an increasing tendency to consider alternatives to reciprocity. For instance, a review on cooperation in animal societies concluded that firm evidence of reciprocity is rare and that alternatives like mutualism, pseudo-reciprocity, and coercion are more important [128].

Mobbing behaviour in birds shows some similarity to alarm calling, in potentially causing a predator to fail to catch prey and to leave the area. Just as in the reasoning about alarm calls in Tr71, there is the view that reciprocity is unlikely as an explanation of mobbing of predators [129]. There are field experiments supporting reciprocity in mobbing between conspecific territorial neighbours in pied flycatchers [130], but there are reasons to consider alternatives. Mobbing of predators that threaten nests is often initiated by highly motivated individuals, like parents with a nearby nest, and subsequent joiners of the mob face less of a risk, as well as a greater benefit of a large mob succeeding in driving the threat away from the area [131]. Also, joiners are often heterospecific birds [131,132], in which case reciprocity would entail surprisingly high cognitive sophistication.

Prey individuals sometimes approach a predator, perhaps to gain information about the nature of the threat, and sometimes this predator inspection is performed jointly by two or more individuals. The interpretation of an experiment with sticklebacks was that predator inspection corresponded to a repeated PDG and that the sticklebacks used the tit-for-tat strategy [133]. This has been debated and, overall, it is not clear that the interaction corresponds to a repeated PDG [134,135].

Food sharing through regurgitation of blood in vampire bats is another much debated case, for which reciprocity has been put forward as an explanation [136,137]. Several factors, such as the helping of relatives and the choice of cooperative partners, might contribute to the evolution of food sharing in vampire bats, and perhaps some form of reciprocity could be one such factor [138], even though the network of interactions between vampire bats does not resemble a repeated PDG. In general, attempting to estimate the relative importance of different suggested evolutionary causes could be a fruitful approach to the study of cooperation.

## 5. Future directions

As our presentation shows, the social sciences have influenced game theory of conflict and cooperation, including applications to non-human animals. This influence is valuable, but comes with a risk of game theory neglecting or not making full use of insights from biology. Among the elements we think should be integrated into game-theory models are accurate representations of life histories, population processes and ecological situations, and in particular, more realistic mechanisms, for instance inspired by animal psychology and neuroscience [139]. The advantages of integration include the potential for closer contact with experiments and fieldwork, and thus an improved capacity to evaluate model predictions. We briefly explain our reasons for the suggestion by summarizing why mechanisms should be integrated into game theory.

### (a) Behavioural mechanisms in game theory

A reason for integrating mechanisms into evolutionary modelling is that the real world is complex. Organisms might not have strategies that are fully optimal in every encountered circumstance, but instead use simpler rules (mechanisms) that perform reasonably well in complex environments [140]. The issue is particularly acute for game theory of social behaviour, where a major aspect of the environment is the decision-making machinery of social partners. The traditional approach in game theory is to assume that individuals have representations of prior distributions of all fitness-relevant aspects of the environment, and use observations to perform Bayesian updates from prior to posterior distributions. The sequential assessment game [37] is an example of such a model of contests between two opponents. In many cases of social interactions, a fully Bayesian approach will be too challenging for modellers to achieve, and it seems likely that it also does not correspond to real organisms, even after much evolutionary fine tuning. In theories of decision making, a distinction is made between ‘small worlds’, which are simple enough for the Bayesian approach to be realistic, and more complex ‘large worlds’, where a decision maker instead needs to rely on simpler rules [141,142]. Integrating mechanisms into game theory is then a large-worlds approach (see [79, chs 5 and 8] for a discussion of this point).

There is some similarity between behavioural mechanisms in animal psychology and neuroscience and ideas about ‘heuristics’ and ‘rules of thumb’ in economic game theory and human psychology. For instance, Tversky & Kahneman [143, p. 1124] write that ‘in general, these heuristics are quite useful, but sometimes they lead to severe and systematic errors’. The general idea has been further developed by Aumann, in a number of lectures and a working paper under the heading of ‘rule rationality’ [144] (see also [145]). Although there is similarity of ideas, one should note that animal psychology and neuroscience are intensely researched fields that provide a wealth of information about mechanisms, such as various forms of learning. This rich source of mechanisms is unique to biology.

The evolution of social dominance in group-living animals shows that mechanism-based large-worlds models can be helpful when exploring previously difficult-to-study phenomena. By implementing behavioural mechanisms similar to reinforcement learning, one can study things like costs of hierarchy formation, winner–loser effects, and bystander effects [79–81], all of which have been studied in fieldwork and experiments. Reinforcement learning has also been used to model aspects of cleaning mutualisms [146].

It is useful to consider two kinds of approaches to game-theory models of conflict and cooperation. One is to drastically simplify the situation, for instance by assuming that there is no variation in individual characteristics. We think this has the severe drawback of eliminating many of the actual phenomena and processes of real interactions. Learning about and responding to variation is an essential and prominent feature of social behaviour [97,147]. Much observed behaviour, such as display and assessment in contests or exploration of the helpfulness of potential partners, only has meaning when there is more than just a little variation in individual characteristics. The other approach is then to allow for such variation and, in cases where Bayesian small-worlds models are too challenging, attempt to implement strategies in the form of realistic mechanisms. Integrating such mechanisms into large-worlds models can still be challenging, but it has the advantage of bringing the model closer to biological reality, thus increasing the contact between theory and observation. These models also provide contact between game theory and general evolutionary biology, by introducing trait evolution, with traits that are components of mechanisms. We think that this is a challenging but promising future for game theory in biology.

## Data Availability

This article has no additional data.
